# Oral and Swallowing Abilities Tool (OrSAT) in Individuals with Type I SMA Older than 24 Months: A Pilot Study

**DOI:** 10.3390/children13060773

**Published:** 2026-06-02

**Authors:** Giulia Stanca, Maria Sframeli, Camilla Verdilio, Beatrice Berti, Lavinia Fanelli, Natalia Longoni, Marisa Maniaci, Giorgia Coratti, Chiara Cutrì, Roberta Onesimo, Concetta Palermo, Daniela Leone, Anna Falco, Giulia Norcia, Valentina Giorgio, Carolina Ausili Cefaro, Antonella Cerchiari, Sonia Messina, Marika Pane, Eugenio Mercuri

**Affiliations:** 1 Pediatric Neurology Unit and Centro Clinico Nemo, Fondazione Policlinico Universitario Agostino Gemelli IRCCS, 00168 Roma, Italy; giulia.stanca@policlinicogemelli.it (G.S.); camilla.verdilio@guest.policlinicogemelli.it (C.V.); beatrice.berti@policlinicogemelli.it (B.B.); lavinia.fanelli@policlinicogemelli.it (L.F.); giorgia.coratti@unicatt.it (G.C.); chiara.cutri@guest.pliclinicogemelli.it (C.C.); concetta.palermo@policlinicogemelli.it (C.P.); daniela.leone@policlinicogemelli.it (D.L.); anna.falco@guest.policlinicogemelli.it (A.F.); giulia.norcia@policlinicogemelli.it (G.N.); marika.pane@policlinicogemelli.it (M.P.); 2 Department of Clinical and Experimental Medicine, University of Messina, 98125 Messina, Italy; msframeli@polime.it (M.S.); natalia.longoni@polime.it (N.L.); marisa.maniaci@polime.it (M.M.); smessina@unime.it (S.M.); 3 Pediatric Neurology, Catholic University of the Sacred Heart—Rome Campus, 00168 Roma, Italy; antonella.cerchiari@opbg.net; 4 Pediatric Unit, Department of Woman and Child Health and Public Health, Fondazione Policlinico Universitario Agostino Gemelli IRCCS, 00168 Roma, Italy; roberta.onesimo@policlinicogemelli.it (R.O.); valentina.giorgio@policlinicogemelli.it (V.G.); 5 Speech Language Pathology Unit, Fondazione Policlinico Universitario Agostino Gemelli IRCCS, 00168 Roma, Italy; carolina.ausilicefaro@policlinicogemelli.it; 6 Management and Diagnostic Innovations & Clinical Pathways Research Area, Neurorehabilitation and Adapted Physical Activity Day Hospital, Bambino Gesù Children’s Hospital IRCCS, 00168 Rome, Italy

**Keywords:** Spinal Muscular Atrophy (SMA), swallowing, dysphagia, oral motor function, OrSAT

## Abstract

**Highlights:**

**What are the main findings?**

- The Oral and Swallowing Abilities Tool (OrSAT), originally developed for infants under 2 years with SMA type I, can also be applied to older children (2–12.6 years), capturing a wide spectrum of bulbar and swallowing function.
- OrSAT scores were associated with the clinical subtype and the level of feeding impairment, from exclusive tube feeding to normal oral feeding.

**What are the implications of the main findings?**

- OrSAT may represent a practical tool for the long-term assessment of bulbar function in treated SMA type I patients beyond infancy.
- Its use may help identify changes in swallowing abilities over time and guide nutritional management and rehabilitative interventions.

**Abstract:**

**Background/Objectives**: The advent of disease modifying therapies (DMTs) for Spinal Muscular Atrophy (SMA) has highlighted the need for reliable tools to assess bulbar function in type I individuals. The Oral and Swallowing Abilities Tool (OrSAT) was originally developed to evaluate swallowing and feeding abilities in infants with SMA type I during the first two years of life. This study aimed to assess the applicability of the OrSAT in a cohort of children with SMA type I older than 2 years. **Methods**: Fifty-two children with genetically confirmed SMA type I, aged 2 to 12.6 years, were included. All participants had received at least one DMT, administered either soon after diagnosis or when treatment became available. Bulbar and feeding abilities were assessed using the OrSAT and results were grouped according to clinical subtype and feeding modality. Given the small sample size of the subgroups and the ordinal nature of OrSAT scores, comparisons between groups were performed using the non-parametric Kruskal–Wallis test. **Results**: At follow-up, 27 children were orally fed, 19 were exclusively tube-fed, and 6 were tube-fed but were also able to eat some food by mouth. The OrSAT scores reflect a wide spectrum of bulbar function from severe to no impairment. Most children who required exclusive tube-feeding at follow-up had already been tube-fed at treatment initiation, while a small number showed improvement in swallowing abilities and the partial recovery of oral feeding during follow-up. **Conclusions**: Our results suggest that the OrSAT, previously used only in the first two years of life, may also be applicable in older children to describe bulbar involvement and monitor changes over time. However, further studies are needed to refine the tool for this age group and to formally validate its use in older children with SMA type I. Its use may contribute to the longitudinal assessment of swallowing abilities and support rehabilitative management.

## 1. Introduction

Spinal Muscular Atrophy (SMA) is a genetic disorder caused by mutations in the SMN1 gene leading to a reduction in SMN protein and subsequent motor neuron loss leading to weakness and muscle atrophy [1]. Classically, SMA has been classified into three main types in childhood, based on clinical severity and age of onset [2]. Type I SMA is the most severe form with onset before the age of 6 months clinically characterized by severe hypotonia and weakness, along with progressive respiratory and bulbar involvement. This includes increasing swallowing and feeding difficulties over time, with swallowing impairments reflecting difficulties in bolus propulsion from the oral cavity to the stomach, and feeding difficulties encompassing impaired oral intake behaviors such as food handling, chewing, and oral preparation. The condition is associated with a survival rate below 8% at 2 years [3,4]. As a result of the involvement of bulbar motor neurons located in the lower brain stem, mouth opening, chewing, swallowing, and speech were invariably affected in type I SMA, with initial signs often present in the first months after birth, and progressive deterioration generally requiring tube feeding and enteral feeding support.

The advent of disease modifying therapies (DMTs) has had a profound impact on both survival and disease progression, including swallowing impairment. Currently available DMTs target the underlying cause of SMA by increasing SMN protein production through different mechanisms. Onasemnogene abeparvovec is a gene replacement therapy delivering a functional copy of the SMN1 gene, whereas nusinersen and risdiplam act on the SMN2 gene through RNA-targeting strategies, using antisense oligonucleotides and small molecules, respectively. By increasing the availability of functional SMN proteins, these therapies can modify the natural history of SMA and significantly improve clinical outcomes [1,2,3,4,5,6,7].

These therapies have produced dramatic improvements not only in survival and motor function, which were the primary measures used in clinical trials, but also in bulbar function, with significant changes in speech and swallowing abilities compared to untreated patients. A reduced risk of developing a severe swallowing impairment and an overall reduction in the signs associated with bulbar dysfunction, compared with the untreated SMA type I population, has been reported both in clinical trials [5,6,7] and real-world data [8,9,10,11]. The risk of developing swallowing abnormalities becomes progressively lower in infants who receive early treatment soon after diagnosis and in those identified before the onset of clinical signs of SMA through neonatal screening [12,13], with a small number of subjects receiving early treatment requiring tube feeding at follow-up.

The advent of DMTs has not only reduced bulbar involvement but has also provided increasing evidence that infants with pre-existing bulbar difficulties may experience partial recovery after treatment [8,9,11]. Recent papers have reported anecdotal cases in which swallowing abilities improved because of the combination of the new DMTs and rehabilitation [8,9,11]. These findings highlight the variability and longitudinal evolution of bulbar function in patients treated with DMTs, including possible changes occurring beyond the first 24 months of life. As survival and clinical stability continue to improve with early treatment, long-term follow-up using appropriate tools for the assessment of bulbar function has become increasingly important to evaluate the sustained effectiveness of therapies over time. In an ideal world all infants with SMA type I should have a structured speech therapy assessment and videofluoroscopy, as highlighted by care recommendations, with regular follow-up assessments. This is not always possible in all centers and there have been recent suggestions to use simple tools that could be used for screening in clinical routines, and identify infants at risk for more detailed assessments [10,11,14].

The OrSAT (Oral and Swallowing Abilities Tool) [14] was specifically designed to record structured information on different aspects of oral, swallowing, and feeding abilities and has been used in both treated and untreated infants with type I SMA [11,14,15,16,17], demonstrating a good sensitivity in detecting the progression of bulbar function across the first 24 months of life.

The aim of this study was to explore the applicability of the OrSAT in older infants and children with SMA type I over the age of 2 years, extending its use beyond the age range originally considered for the tool. Given the increasing survival of patients treated with DMTs and the evolving clinical course of bulbar function, we sought to investigate whether the OrSAT could provide meaningful longitudinal information in this older population. More specifically, we analyzed swallowing abilities before and after DMT treatment and explored the possible differences in OrSAT scores according to SMA type I subtype, functional status, and the need for tube feeding at follow-up.

## 2. Materials and Methods

### 2.1. Subjects

This retrospective observational study is part of a larger natural history study on SMA approved by the Institutional Ethics Committee (2355/18 ID: 1894). The parents of the participants provided fully informed consent.

The cohort included 52 symptomatic children with type I SMA (22 females and 30 males), aged 2–12.6 years, who underwent routine follow-up assessments for neuromuscular monitoring at participating centers between February 2023 and May 2025. These evaluations were performed in different clinical settings, including day-hospital visits, scheduled hospital admissions, and study-related assessments. All children had a genetically confirmed diagnosis of SMA and a clinically confirmed diagnosis of type I SMA.

They were all treated with nusinersen, onasemnogene abeparvovec (OA) or risdiplam administered either as single or combined therapies, shortly after diagnosis or, in the older individuals, as soon as these treatments became available.

In order to assess overall clinical severity, the cohort was classified according to the Dubowitz’s decimal classification: 1.1, the more severe end of the spectrum with severely reduced mobility and early respiratory and bulbar involvement; 1.5, the most common phenotype, with inability to raise the legs against gravity but no overt feeding or respiratory difficulties at diagnosis; 1.9, the mildest phenotypes, infants may achieve head control [2,18,19].

Prior to the initiation of DMTs, the feeding method of each child was documented. Specifically, it was noted whether they ate exclusively by mouth, whether they had a G-tube but could still eat some food by mouth, or whether they were fed entirely via enteral nutrition.

Following the initiation of DMTs, all subjects, including individuals who received DMTs at a later age, were evaluated using the OrSAT checklist to assess the progression of their swallowing abilities over time. As soon as the OrSAT checklist was developed, it was administered at the patients’ first routine follow-up visit for their underlying disease. Follow-up visits were scheduled according to routine clinical practice and varied depending on the patient’s clinical condition, the specific DMT administered, and participation in clinical trials, generally occurring every 4 or 6 months.

The Kruskal–Wallis statistical test was used to analyze the total score according to SMA subtype.

### 2.2. OrSAT

The OrSAT includes a checklist consisting of 12 structured questions addressed to the main caregiver assessing individual aspects of swallowing [14].

In the first four items, the checklist is designed to investigate the child’s capacity to consume foods of varying consistencies, namely liquid, semi-liquid, semi-solid, and solid. Other items explore whether postural adjustments or other adjustments, such as the need to alter the texture of foods by blending or thickening them, are needed to make mealtimes safer. The checklist also includes questions about coughing or pharyngeal residue during mealtimes, the child’s fatigue levels related to eating, or the ability to finish the meal, including information about the duration of the meal.

The last two items explore the ability to produce syllables in the first months or clear words.

Each item is scored based on whether the child is able (1) or unable (0) to perform the activity. A higher score represents a higher level of oral motor feeding function.

A separate part of the OrSAT allows us to identify four descriptive levels of impairment. The functional levels are scored separately from the checklist and include: “Severe impairment” (level 1)—unable to swallow by mouth, requiring tube feeding. Intermediate levels include “Moderate impairment”—able to swallow some food consistencies safely but need oral supplements or tube feeding (level 2); “Mild impairment”—safe swallowing but requires compensatory strategies or other intervention (level 3), and “No impairment” (level 4)—safe and efficient swallowing for all consistencies. The number of choices allows us to identify the level that is consistent with what is reported by the carers.

The OrSAT is performed as a structured interview addressing the main caregiver as part of clinical routine assessments. At the beginning of this study, most patients had been on treatment for variable amounts of time (see previous paragraph). In this cohort the OrSAT was administered at the first assessment after the scale became available.

Similar to the procedure used in the development of the OrSAT in younger infants, the scale was performed in a sample (*n* = 78) of typically developing children between the ages of 2 and 12 years, used as an age-matched control group within the original OrSAT framework. Consistent with what was observed in the cohort between 1 and 2 years, more than 95% of the children had full scores on both the individual items and on the overall OrSAT score, with a very high intra-rater concordance (K = 0.89).

## 3. Results

A total of 52 individuals with type I SMA were included in the study. Their ages ranged between 2.0 and 12.6 years. At baseline, i.e., before the start of DMTs, 27 were orally fed, and 25 were tube fed; of these, 19 were exclusively tube fed while the remaining 6 were also able to receive some food by mouth (mixed feeding). They all showed weakness related to SMA at the time of diagnosis.

All were treated with at least one of the available DMTs: nusinersen (*n* = 24/52), OA (*n* = 10/52), risdiplam (*n* = 2/52) as a single treatment or switching from nusinersen to OA (10/52). Another 6 individuals had OA combined with nusinersen (1/52) or risdiplam (5/52). Treatment was administered early after diagnosis or, in the older individuals, as soon as it became available. The age at treatment initiation ranged between 1 and 68 months (mean: 14 months, SD: 18 months), with 23 of the 52 children receiving treatment before the age of 6 months.

### 3.1. OrSAT

The OrSAT scores ranged between 0 and 12. The levels ranged between severe and no impairment. They were all showing weakness related to SMA at the time of diagnosis. The data obtained using the OrSAT checklist were analyzed by stratifying the patients according to clinical subtype (1.1, 1.5 and 1.9) and feeding modality, identifying individuals who were exclusively fed orally, and those who were tube fed, using percutaneous endoscopic gastrostomy (PEG), providing details if they were exclusively tube fed or were still able to receive some food by mouth.

### 3.2. SMA I Subtype, OrSAT Scores, Functional Levels, and the Need for Tube Feeding at Follow-Up

The 1.1 cohort included 7 individuals (all males; age range: 2.5–7.4 years). At follow-up 4/7 (57.1%) were tube-fed and classified under ‘severe impairment’ on the functional levels. Their total score on the OrSAT checklist ranged between 0 and 1. The remaining three individuals (42.8%) were orally fed and classified as “Mild Impairment” (1 subject) or “No Impairment” (the 2 remaining individuals). Their total score on the OrSAT checklist ranged between 8 and 11.

The 1.5 cohort included 34 individuals (16 females, 18 males; age range: 2–12 years). At follow-up 15 (44%) were orally fed and had a functional level of “Mild Impairment” (3/15, 20%) or “No impairment” (12/15, 80%). Their total score on the OrSAT checklist ranged between 7 and 12. Five of them (5/34, 15%) were tube-fed but were also able to receive some food by mouth and showed a functional level of “No impairment” (1/5, 20%), “Mild impairment” (2/5, 40%), or “Moderate Impairment” (2/5, 40%). Their total score on the OrSAT checklist ranged between 4 and 9. The remaining 14 individuals (41%) were exclusively tube fed and had a functional level of either “Severe impairment” (7/14, 50%) or “Moderate Impairment” (7/14, 50%). Their total score on the OrSAT checklist ranged between 0 and 2.

The 1.9 cohort included 11 individuals (6 females and 5 males; age range: 4.5–12.8 years). At follow-up 9/11 of them (81%) were orally fed and showed a functional level of either “No impairment” (8/9 subjects, 89%) or “Mild Impairment” (1/9, 11%). Their total score on the OrSAT checklist ranged between 7 and 12. One other individual, who was primarily tube-fed but still able to consume some food orally, had a functional level of “Moderate impairment” and a total score on the OrSAT checklist of 4. The last individual was exclusively tube fed and showed a functional level of “Moderate impairment” and a total score on the OrSAT checklist of 2.

**Figure A1:**
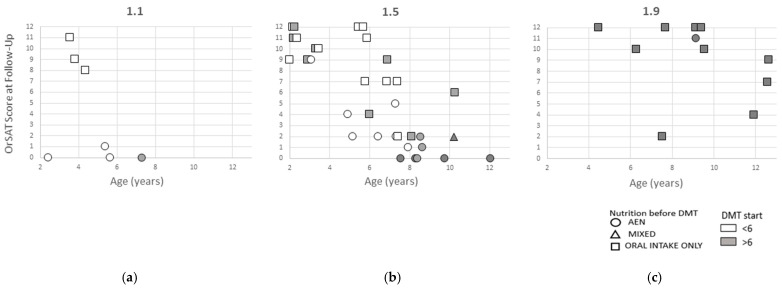
(**a**–**c**). Distribution of OrSAT scores at follow-up according to age at treatment (months) and baseline nutritional status, stratified by SMA type I subgroups: type 1.1 (**a**), type 1.5 (**b**), and type 1.9 (**c**). Each panel illustrates relationship between treatment timing and swallowing functional abilities, as measured by OrSAT, across different nutritional conditions at baseline.

Figure A1a–c provides details of the OrSAT scores at follow-up while also providing details of age at treatment and nutrition status at baseline.

### 3.3. Feeding Status at Follow-Up and Need for Tube Feeding at Time Treatment Started

Among the 27 individuals (51.9%) who were orally fed at follow-up (range: 13–139 months, mean: 66.4 months, SD: 28.73), all but one were already orally fed at the time of DMT initiation. The remaining subject transitioned from tube feeding to complete oral feeding during follow-up.

Of the 6 individuals who were tube-fed but were also able to eat some food by mouth at follow-up, three were exclusively tube-fed at baseline and had an improvement in their swallowing abilities between baseline and follow-up (range: 1–117 months, mean: 62, 35; ds: 27). Three additional subjects, initially exclusively orally fed, required subsequent transition to tube feeding: one for swallowing difficulties, one for poor weight gain and perioperative nutritional optimization before spinal stabilization, and one for mixed bulbar dysfunction and insufficient growth.

Of the 19 individuals who were exclusively tube-fed at follow-up (range: 28–127 months; mean: 69.91; ds: 23.74), 15 (79%) were already receiving exclusive enteral nutrition prior to the initiation of DMT, one (5%) was primarily tube-fed but still able to consume some food orally (via PEG and oral intake), and three (16%) were exclusively orally fed at baseline. In one case, a percutaneous endoscopic gastrostomy (PEG) was inserted because of esophagitis and stenosis, which made continuing oral feeding impossible. Two other children had a PEG tube inserted at a later age after their families made decisions not recommended by doctors. In one case, the child was fed for a long time through intermittent tube feeding before the decision to perform a PEG was taken. The other individual discontinued oral feeding after an acute chest infection requiring orotracheal intubation (IOT). After this acute event parents refused to restart oral feeding because of fear of recurrence, despite safe swallowing on the clinical observation. Overall, these cases represent heterogeneous external factors (gastrointestinal complications, acute respiratory events, and caregiver-driven decisions) influencing feeding route, rather than a consistent deterioration of bulbar function.

**Figure 1:**
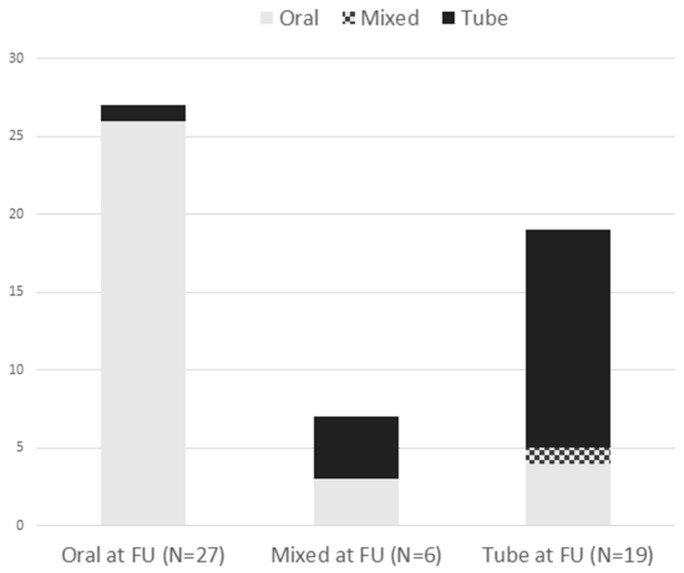
Feeding Status at the Start of DMTs and Follow-Up.

The need for tube feeding was consistent between treatment initiation and follow-up in 79% of cases, whereas 96% of patients who were orally fed at baseline remained orally fed at follow-up (Figure 1).

The heterogeneity in follow-up duration reflects the observational nature of the study and the variability in clinical follow-up schedules, which in SMA practice are not uniform and typically range from 4 to 6 months depending on patient condition, treatment regimen, and participation in different therapeutic programs. This variability should be considered when interpreting longitudinal outcomes.

**Figure 2:**
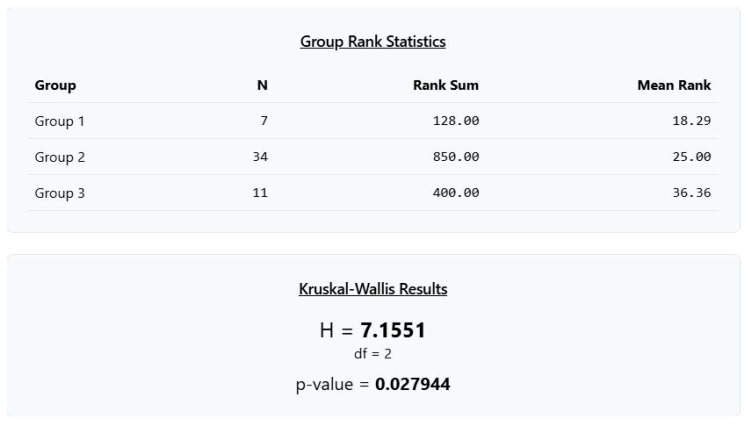
Kruskal–Wallis test. Group 1 = SMA 1.1; group 2 = SMA 1.5; group 3 = SMA 1.9.

A Kruskal–Wallis test was conducted to examine the differences between three groups. The analysis revealed a statistically significant difference among the groups, where H (2) = 7.16 and *p* = 0.028 for both SMA type and feeding modalities (Figure 2).

## 4. Discussion

The OrSAT has been increasingly used in clinical practice and in research studies due to its ability to quantify different degrees of bulbar involvement in young infants with SMA [10,11,14,20]. The scale, originally developed as a tool to assess very young infants and to follow the development of more advanced swallowing functions, has been validated for infants until the age of 24 months [14]. With the increased survival of treated infants with type I SMA, emerging evidence indicates that bulbar function may also change beyond 24 months, including both deterioration requiring tube feeding and, in some cases, the partial recovery of oral feeding abilities, particularly in individuals treated early compared to untreated historical cohorts [8,20]. These findings have highlighted the need for the continued monitoring of bulbar function beyond infancy. In this study, we explored the applicability of the OrSAT in children older than 24 months with SMA type I. Our findings suggest that the OrSAT retains the ability to capture a broad spectrum of oral and bulbar functional abilities in this age group, although further adaptation and formal validation are required. While a small pilot study of typically developing children showed that they all achieved a full score on the scale, its application in children with type I older than 24 months showed a wide range of scores that were associated with different levels of swallowing abilities and/or a partial or full-time need for tube feeding. Children requiring full-time tube feeding generally had checklist scores of two or below, mainly driven by the speech-related items rather than swallowing performance. These items, initially included when treated children with type I began to demonstrate the ability to produce clear words and to capture the onset of this ability, are however less relevant for older children and not specific for the assessment of oro–bulbar function in this age range.

As previously observed in younger children, the magnitude of OrSAT scores and different levels were broadly consistent with disease severity, increasing progressively from type 1.1 to 1.9 [10,11]. However, this observation should be interpreted cautiously due to the small size of subgroup 1.1.

Regarding feeding outcomes, most children requiring tube feeding at follow-up were already tube fed at the time of treatment initiation while only five required tube insertion during follow-up. Interestingly, in two of these cases, feeding changes were often associated with non-neurological factors, such as gastrointestinal complications or intercurrent medical events, suggesting that changes in feeding status do not necessarily reflect worsening bulbar function.

Our results also confirm and expand previous observations that swallowing difficulties at baseline are largely associated with bulbar outcome [10] as the concordance between feeding status at the time of treatment and at follow-up was 79%. These longitudinal observations should be interpreted in the context of the observational design of the study and the inherent variability in clinical follow-up schedules in SMA, depending on patient condition, treatment regimen, and clinical program participation, leading to heterogeneous cumulative observation times across patients.

The discordant findings were not always toward a deterioration of bulbar activity. In four cases, in contrast, improvements in oral feeding ability were observed during follow-up in the context of speech and language rehabilitation support programs, including oral stimulation, postural control, and strategies aimed at reducing functional limitations, with three children regaining partial oral feeding and one transitioning from enteral nutrition prior to treatment to exclusive oral feeding. However, given the observational nature of the study, these findings should be interpreted as descriptive associations rather than evidence of treatment or rehabilitation effect. Conversely, deterioration in swallowing function was observed in seven individuals, with only one of them switching to exclusive tube feeding at follow-up (OrSAT score = 2).

These results are at variance with the progressive impairment reported in natural history studies [3]. However, given the absence of a contemporaneous untreated control group, these comparisons should be interpreted as indirect and contextual rather than as direct comparative evidence. Overall, the results are consistent with a more favorable clinical course.

The variability in progression within this patient group also highlights the need for follow-up over time. Even when limited to a relatively small group, our findings suggest that the OrSAT may capture a broad spectrum of oral functional abilities in children over two years of age with SMA type I. Importantly, this study was not designed as a formal validation study of the OrSAT in children older than 24 months, but rather as an exploratory assessment of its applicability in this population. Therefore, findings should be interpreted accordingly, and dedicated validation studies are required before clinical implementation beyond infancy. The current version is therefore not yet optimized for use beyond infancy, and formal validation in this population is required.

In line with these limitations, we are currently developing an adapted version of the OrSAT specifically designed for older children and for broader SMA subtypes which will undergo formal validation in future studies. As previously discussed, modifications and adaptations are needed to improve this screening tool’s specificity in detecting bulbar function and informing the choice of the most appropriate rehabilitative intervention.

## Figures and Tables

**Figure A2:**
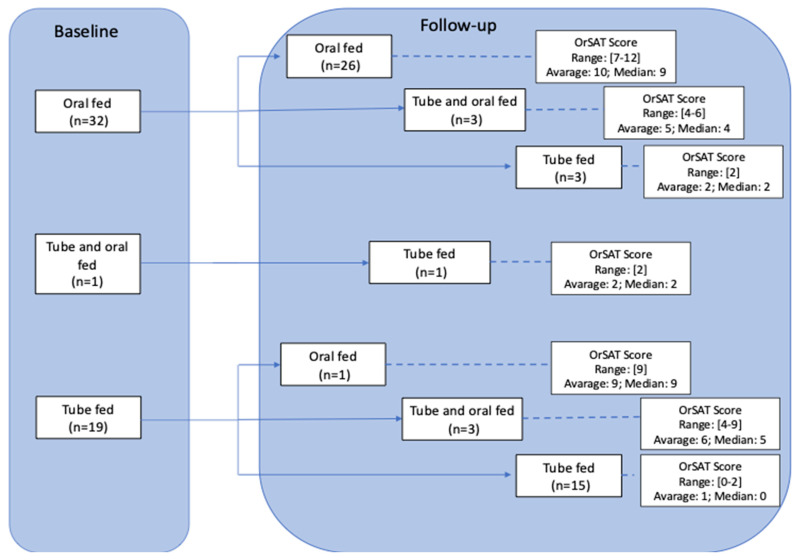
Evolution of feeding modalities from baseline to follow-up and corresponding OrSAT scores. At baseline, patients were classified as oral (*n* = 32), mixed (*n* = 1), or tube feeding (*n* = 19). Arrows indicate transitions at follow-up. Most orally fed patients remained oral (*n* = 26; OrSAT 7–12), while some shifted to mixed (*n* = 3; 4–6) or tube feeding (*n* = 3; 2). The mixed baseline case transitioned to tube feeding (*n* = 1; 2). Among tube-fed patients, one became oral (*n* = 1; 9), three mixed (*n* = 3; 4–9), and most remained tube-fed (*n* = 15; 0–2). Values are reported as range (mean and median shown in figure).

## Data Availability

The data presented in this study are available on request from the corresponding author. The data are not publicly available due to privacy and ethical reasons.

## Abbreviations

The following abbreviations are used in this manuscript:

SMA	Spinal Muscular Atrophy
DMT	Disease modifying therapies
PEG	Percutaneous Endoscopic Gastrostomy
IOT	Intubation and Oral Tracheal ventilation

## Appendix A

**Table A1:** Baseline characteristics of the cohort.

	Whole Cohort (N = 52)	No Need for Tube Feeding (N = 32)	Tube Feeding and Oral (N = 1)	Exclusively Tube Feeding (N = 19)
Age (months)				
Mean (SD)	14.1 (18)	12.6 (18.5)	10.2 (-)	15.3 (17.1)
Median [range]	6 [1–68]	6 [1–68]	10.2 [10.2]	5 [1–65]
Gender				
F, n (%)	22 (42)	17 (53)	1 (100)	4 (21)
M, n (%)	30 (58)	15 (46)	0 (0)	15 (79)
SMA subtype				
1.1, n (%)	7 (13)	3 (9)	0 (0)	4 (21)
1.5, n (%)	34 (65)	19 (60)	1 (0)	14 (74)
1.9, n (%)	11 (21)	10 (31)	0 (0)	1 (5)
Tube feeding				
Yes, n (%)	34 (65)	0 (0)	0 (0)	19 (100)
No, n (%)	18 (35)	32 (100)	1 (0)	0 (0)
Treatment				
Nusinersen, n (%)	24 (48)	12 (38)	1 (100)	11 (58)
OA, n (%)	10 (17)	9 (28	0 (0)	1 (5)
Risdiplam, n (%)	2 (2)	2 (6)	0 (0)	0 (0)
Switch				
Nus. + OA, n (%)	10 (19)	6 (19)	0 (0)	4 (21)
Nus. + Risd, n (%)	0 (8)	0 (0)	0 (0)	0 (0)
OA + Nus., n (%)	1(4)	1 (3)	0 (0)	0 (0)
OA +Risd, n (%)	5(2)	2 (6)	0 (0)	3 (16)

### Levels of Impairment

**Table A2:** Descriptive levels of impairment OrSAT.

Levels of Impairment			
	Scores 0–5 m	Scores 6–9 m	Scores 10–24 m
Severe: individual is not able to swallow anything safely by mouth. All nutrition and hydration is received through non-oral means (e.g., nasogastric or G-tube).			
	0–1	0–1	0–1
Moderate: swallowing for thin liquids is safe but the infant gets easily tired and unable to complete a full meal and takes less than 50% of nutrition and hydration by mouth. These children may require need for oral supplements or alternative method of feeding (NG tube or G-tube).			
	2–3	2–3	2–4
Mild impairment: swallowing is safe, but usually requires moderate cues to use compensatory strategies or more careful posturing or other intervention (thickening food).			
	4–5	4–6	5–8
No impairment: the individual’s ability to eat is not limited by swallow function. Swallowing is reported as safe and efficient for all consistencies (when age appropriate), without choking episodes or other clinical signs such as retching or cough.			
	6–7	7–10	9–12
